# Supplementary material for: Are carbon emissions trading and green financial instruments synergistic? -Comprehensive quantitative research based on content analysis
Source: PLoS One. 2024 Mar 7;19(3):e0298601. doi: 10.1371/journal.pone.0298601 (PMC10919601; doi:10.1371/journal.pone.0298601)
Supplement: S1 Data — (ZIP) [file pone.0298601.s001.zip › Data/地级市Z_F工作报告.zip!/┴╔─■/╕╖╨┬╩╨/└┤╫╘╣1⁄2╓┌║┼í╛┬φ┐╦╩2╛▌═°í┐_2018.docx]

各位代表：

现在，我代表市政府向大会报告工作，请予审议，并请市政协委员和其他列席同志提出意见。

一、过去五年工作回顾

过去五年，在习近平新时代中国特色社会主义思想指导下，在省委、省政府和市委的正确领导下，在市人大依法监督和市政协民主监督下，全市广大干部群众认真践行新发展理念和“四个着力”“三个推进”，坚持稳中求进工作总基调，攻坚克难，砥砺前行，经济企稳回升，社会全面进步，转型升级进入新阶段。

——转型振兴步伐更加稳健。全面落实中发7号和国发28号、62号文件精神，新一轮东北振兴战略政策落地见效。我市被纳入国家产业合作与转型升级示范区规划；清河门区列入国家采煤沉陷区综合治理试点；新邱区列入国家新增独立工矿区改造搬迁资金支持范围。接续替代产业培育、基础设施建设、生态环境保护等工作取得明显进展，转型之初面对的就业难、住房难、行路难、就医难、就学难、生活难等问题逐一化解，基本完成转型初期确定的任务。

——经济发展基础更加坚实。确立了工业“344”产业布局，大力实施“中国制造2025行动纲要”，装备制造、农产品加工、能源三大支柱产业占全市工业比重达到70%以上。沈阜200万亩现代农业示范带初步建成，示范带内76.3万农业人口人均增收8655元；畜牧业强市战略深入实施。服务业对经济的拉动作用增强，新增上市企业2户、金融机构20家。重大项目建设成效显著，保利、大唐、华能、华录等20多户央企在阜投资，同江苏徐工、中国航天、上海宝钢合资合作进展顺利，富国皮革、天佐羊绒、大鹰水泥等总部搬迁落户阜新。我市被命名为国家新能源示范市，经济开发区晋升为国家级高新区。省中小微企业创业基地、新型材料产业基地晋升为省级开发区。

——改革动能释放更加充分。“放管服”改革纵深推进，市级行政审批事项减少564项，非行政许可审批事项全部取消，市级议事协调机构和临时机构撤销466个，机关公务用车制度改革全面完成。交投、城建等企业集团组建，123个经营性事业单位转制。建立政府偿债准备金、养老保险风险基金并完善相关制度。取消、停征、调整54项行政事业性收费和政府性基金项目，减免企业税费近3亿元。我市被列为全国4个工业用地市场化配置改革试点市之一。不动产统一登记、国有林场等改革扎实推进。制定出台了重大行政决策程序等20多项政策措施，政府法律顾问实现全覆盖。

——城乡面貌变化更加明显。

新一轮城市总体规划获批，常住人口城镇化率达到58.4%。开通阜盘、彰通2条高速公路，高速公路总里程居全省前列；巴新铁路接入国家铁路网，京沈高铁阜新段及彰武联接线线下工程完工；“锦阜高”铁路扩能改造部分通车；新邱火车站改造完成；新建维修城区道路37条，农村实现屯屯通油路，城市大外环通车。完成供水、供热、供气等主管网改造，实现自来水从源头到龙头全程监控，天然气管网主城区全覆盖。500千伏输变电及配套工程加快推进。

——生态环境更加宜居。完成主城区化工、铸造企业搬迁，拆除燃煤小锅炉700多台，淘汰黄标车及老旧车辆2.5万辆。市级饮用水水源、水质达标率均为100%，日污水处理能力达到28.5万吨。城区地下水源井全部关闭。累计人工造林203.7万亩，城区绿化覆盖率达到41.1%。草原沙化治理72万亩。海州露天矿灾害治理5000万欧元德国贷款获批。高德矸石山综合治理等生态项目竣工。细河全流域治理提升、城市防汛“1+3”、宜居乡村等重点工程稳步推进。

——民生保障更加有力。累计实名制就业22.5万人。投入30多亿元资金，保障了近20万困难群众基本生活。改造棚户区300多万平方米、惠及10万多人。获批国家扶贫改革试验区。教育、卫生、文化、体育、养老等一批民生实事按期办结。农村无害化卫生厕所普及率达到50.9%。万人坑死难矿工纪念馆改造完成。广播剧《好大一棵樟子松》、电影《谁来陪伴我》获国家级大奖。荣获“世界玛瑙之都”称号。成功创建全国双拥模范城、全民健身示范市。彰武县成功创建国家级卫生县城。

各位代表，刚刚过去的2017年是本届政府的收官之年。这一年，我们积极应对经济下行压力加大、各种风险挑战增多的严峻形势，统筹推进稳增长、促改革、调结构、惠民生、防风险等各项工作，基本完成了市十五届人大六次会议确定的目标任务。预计地区生产总值417亿元，增长0.1%；一般公共预算收入38.2亿元，增长6.5%；固定资产投资110亿元，增长23.6%；出口总额15亿元，增长12%；城镇和农村常住居民人均可支配收入分别达到25460元、12520元，分别增长6.2%、6%。一年来，主要抓了以下几方面工作。

（一）全力抓好项目建设，促进实体经济发展

“项目年”“招商季”“服务月”活动扎实推进，“两个牵动、四个一百”工程取得实效。大唐煤制天然气项目债务重组和产品方案优化进展顺利。页岩气勘探开发取得积极成效。新开工五千万元以上项目105个、竣工投产项目103个、推进建设项目107个，新增亿元以上储备项目114个。坚持领导带队招商、点对点招商、上门招商，推进环保产业园、义乌小商品城等项目取得实质进展，上海三爱富化工、三一重工产业园等项目达成意向。内资到位77亿元，增幅走在全省前列；直接利用外资2.4亿美元，增幅居全省首位。组建市营商环境建设监督局，容缺审批、承诺制、协同代办等制度全面推行。

（二）着力深化改革创新，为发展提供强劲动力

供给侧结构性改革扎实推进，“三去一降一补”任务全面完成。商品房销售面积73.5万平方米，增长12%；全面排查企业债务金融风险，确保总体可控；认真兑现涉企优惠政策，帮助企业降成本近亿元；逐步补齐县域经济、生态、人才等短板。建立了“9110”制度，明确了“3550”时限目标，行政审批效率明显提升。启动“多规合一”“多证合一”改革，新增市场主体1.5万户。优化调整40个政府部门和单位的“三定”方案。阜新发电公司整合城区主要热源完成，虎跃集团重组市公交公司，210台新能源公交车上线运营。中央及其下放企业“三供一业”分离移交工作进展顺利。科技创业创新彰显活力，高新技术产品增加值占规上比重20%，实施科技成果转化31项，净增高新技术企业14户。

（三）努力稳定工业增长，推动产业转型升级

重点产业进一步优化升级。德尔变速箱油泵研发等11个项目列入省技术创新计划，鲁花扩能、伊利技改等项目正在建设，凯莱英医药等项目竣工投产，新开工光伏发电项目27万千瓦、风电10万千瓦。深化工业园区体制机制改革，园区基础设施投资增长10.1%，皮革产业开发区晋升为中国轻工业特色区域和产业集群创新升级示范区。春成工贸通过国家首批“两化”融合试点验收。万达公司高铁机车联系枕梁产品填补国内空白。全年新晋规上企业26户。

（四）大力推进结构调整，加快发展现代农业

现代农业示范带三年滚动计划开局良好，巩固提升67万亩，新建36万亩。调减玉米种植面积21.3万亩，粮食产量39.8亿斤。新建改扩建标准化养殖小区27个。建成高标准农田18.4万亩。新增节水滴灌8.6万亩。实现大旱之年减产未减收，全市农村呈现出人心稳、信心足的良好局面。玉米耕种收综合机械化率达到89.4%。秸秆综合利用率达到85%。农村土地承包经营权确权工作走在全省前列，两县共确权737万亩，溢出土地120多万亩。阜蒙县、彰武县分别成为国家农村三产融合、集体产权制度改革试点县。十家子镇入选国家级特色小镇，章古台镇等7个乡镇成为省级特色乡镇，王府镇烟台营子村被命名为国家级少数民俗特色村寨。

（五）致力提高服务业档次，促进新兴业态发展

服务业布局加快，兴隆CBD三期开工，万达广场、红星美凯龙项目主体封顶，集聚区吸纳效应初步显现；华唐服务外包基地、国家生猪交易市场等电子商务项目运营，中国邮政集团物流中心项目签约。金融机构新增贷款87亿元，增长10.4%，营口银行阜新分行开业运营，阜新银行、金凯化工上市工作进展顺利。6个省级服务业集聚区营业收入338亿元，增长10%。旅游总收入实现90亿元，增长12%。社会消费品零售总额290.6亿元，增长3%。主动融入“一带一路”战略，对俄罗斯出口增长6倍。

（六）强力抓好环境治理，优化城乡生态条件

全力配合中央环保督察，258个案件全部按时办结，反馈的12个问题整改取得阶段性成果。开展大气扬尘污染防治百日攻坚战，秸秆焚烧防控成效明显，拆除燃煤锅炉353台、淘汰黄标车及老旧车辆4002辆，超额完成省定任务。全年环境空气质量达标293天。全面推行市、县、乡三级河长制。土壤环境质量监测网络初步建立。辽西危险废物处置中心通过验收。完成人工造林16万亩，东新大街等22条街路新植补植树木14.8万株。集中修补人行道板1.1万平方米。改造供水、供热、供气等地下管网58公里。宜居乡村建设扎实推进。

（七）倾力保障改善民生，提高人民群众幸福感

脱贫攻坚创造阜新经验，全省扶贫现场会在我市召开。28980名贫困人口脱贫，73个贫困村销号。新增城镇就业1.65万人。社会保障各类提标扩面全部兑现。棚户区改造新开工1291套，改造农村危房2363户。改造薄弱学校18所，维修校舍33万平方米。细河区和清河门区通过国家义务教育均衡达标认定。24家公立医院实行改革，药品加成全部取消。在第十三届全运会上，我市运动员取得7金3银4铜。文艺创作繁荣，进省进京巡演巡展10余次。安全生产形势总体平稳。社会治安防控体系日益完善。民族团结进步事业不断发展，宗教领域和谐稳定。食品药品监管水平提升。人防工作取得新成效。政府债务、企业养老金、重点企业信贷风险有效防范。

一年来，我们深入学习贯彻落实党的十八大、十九大精神，深入推进“两学一做”学习教育常态化制度化，严格执行中央八项规定精神和省市委实施细则，重点领域、关键环节的行政监察和审计监督力度加大。认真做好政府立法工作。坚持依法科学民主决策，自觉接受市人大依法监督、市政协民主监督和社会各界监督，高度重视人大代表建议、政协委员提案办理。“七五”普法全面实施。

全市各行各业、各条战线都呈现出新变化，外事、侨务、对台、市志、档案、工会、青年、妇女儿童、残疾人事业、红十字、老龄、慈善等工作取得新成绩，国税、地税、消防、电力、邮政、通信、石油、烟草等单位为地方经济发展作出了积极贡献。

各位代表，经过本届政府五年的努力，我们走出了一条经济与社会协调并进、总量增长与结构调整良性互动的转型升级有效路径，为今后更长时期发展奠定了坚实的基础。成绩来之不易，这是党中央、国务院亲切关怀和省委、省政府正确领导的结果，是市委总揽全局，领导全市人民齐心协力、顽强拼搏的结果。在此，我代表市人民政府，向全市各族人民，向市人大代表、政协委员和各民主党派、工商联、人民团体、无党派人士，向离退休老领导、老同志，向驻阜中省直单位、部队指战员、武警官兵和社会各界人士，向所有关心、帮助、支持阜新发展的同志们、朋友们，表示衷心的感谢并致以崇高的敬意！

各位代表，阜新转型振兴进入新阶段，站在新的历史起点上，我们清醒地认识到，我市经济社会发展还面临不少困难，政府工作还存在一些问题。经济发展不充分、经济总量不足、产业结构不优是阜新最大的市情；经济增长的基础还不牢固，产业规模不大，重大项目不多，实体经济发展困难；财政收支矛盾十分突出，生态环境保护治理任务更加繁重，安全生产、防范风险压力依然很大，保障改善民生面临不少难题；少数政府工作人员担当精神和执行力还有差距，政府服务和机关作风还需进一步改进。对这些问题，我们一定高度重视，采取有力措施切实加以解决，努力把政府工作做得更好，决不辜负人民群众的期望！

二、今后五年主要目标任务

新一届政府的五年，是我市全面建成小康社会，推进新阶段阜新转型升级的关键时期。阜新转型经历了三个阶段：第一阶段是2001—2005年，为转型起步阶段。第二阶段是2006—2012年，为转型推进阶段。第三阶段是2013—2020年，为转型升级阶段，这个阶段分为两个时期，我们已经走过了攻坚期，进入了决胜期。站上新起点，踏上新征程，我们必须凝聚力量，奋发有为，加快推动阜新全面转型升级，确保如期全面建成小康社会。

今后五年政府工作的总体要求是：以习近平新时代中国特色社会主义思想为指导，深入学习贯彻党的十九大精神，坚持稳中求进工作总基调，坚持以供给侧结构性改革为主线，牢牢把握高质量发展要求，统筹推进“五位一体”总体布局和协调推进“四个全面”战略布局，持之以恒落实新发展理念和“四个着力”“三个推进”要求，坚定不移贯彻落实习近平总书记“积极探索资源枯竭型城市转型发展新路子，努力形成活力迸发的新增长区”的指示精神，按照省委、省政府“一带五基地”建设和“五大区域发展战略”总体部署，以推进转型升级为着力点，打好防范化解重大风险、精准脱贫、污染防治三大攻坚战，统筹推进稳增长、促改革、调结构、惠民生、防风险各项工作，全力推动经济社会向更高水平发展，为实现阜新全面转型升级目标而努力奋斗。

今后五年，全市经济社会发展目标分两个时段安排，第一时段，到2020年，实现突破辽西北、县域经济三年计划目标，完成经济转型试点任务，与全省一道全面建成小康社会；第二时段，到2022年，巩固提升转型成果，努力实现生产发展、生活富裕、生态良好，朝着新阶段全面转型升级目标迈进。

主要指标预期是：经济发展速度不低于全省平均水平，到2022年，地区生产总值年均增长6.5%，规上工业增加值年均增长10%，一般公共预算收入年均增长6.5%，固定资产投资年均增长15%，城乡居民收入增长与经济发展实现同步。

围绕市十二次党代会确定的奋斗目标，推进“三个升级”取得新成效：

——推进经济结构优化升级，建设充满活力的阜新。推动经济发展质量变革、效率变革、动力变革，接续替代产业壮大，多元化产业体系形成。规模以上工业企业达到300户以上，主导作用进一步凸显。现代农业集约化、规模化、标准化、产业化水平显著提高，基础地位更加巩固。现代服务业占比进一步提高，消费对经济增长的贡献明显加大。

——推进发展动力转换升级，建设开放创新的阜新。供给侧结构性改革扎实推进，重要领域和关键环节改革取得重要成果，科技创新、产业创新、人才创新能力明显提高，核心竞争力进一步增强。对外开放水平大幅提升，引进内资、外资和出口总额分别年均增长15%、15%、5%以上。产权有效激励、要素自由流动、竞争公平有序、企业优胜劣汰的营商环境形成。

——推进社会建设提质升级，建设幸福美丽的阜新。社会保障体系进一步健全完善，公共服务进一步均等化，社会事业全面进步。城市布局更加合理，功能更加完善，数字阜新、智慧阜新取得积极进展。生态文明建设成效显著，环境质量明显改善，森林覆盖率达到30%，环境空气质量达标天数保持在80%以上。社会治理法治化水平全面提升，形成人民安居乐业、社会和谐稳定的良好局面。

三、2018年工作安排

今年是贯彻落实党的十九大精神的开局之年，也是新一届政府的开局之年。做好今年工作，必须认真贯彻落实市委十二届八次全会精神。全市经济社会发展的主要预期目标是：地区生产总值增长6.5%；规上工业增加值增长7%；一般公共预算收入增长6.5%；固定资产投资增长15%；城镇和农村常住居民人均可支配收入均增长6.5%；城镇登记失业率控制在5%以内，完成省下达的资源环境约束性指标。为实现上述目标，重点做好以下十项工作：

（一）以项目建设为核心，巩固经济稳中向好势头

牢固树立抓项目就是抓有效投资、抓发展、抓后劲的理念，把扩大有效投资作为稳增长的首要任务，坚决打好投资攻坚战。

实施“3215项目牵动计划”，以大唐煤制天然气、页岩气勘探开发、富国皮革项目建设为牵动，全年新增亿元以上储备项目200个，新开工投资五千万元以上项目150个。着力推进央企合作、战略重组、产业协作、科技嫁接、总部经济等拉动性大、支撑性强的大项目，形成一个项目带动一个产业。

全力推进大唐煤制天然气项目继续建设。油气勘探开发合作实现新突破。加快富国皮革产业链和“零排放”、保利机器人生产线、彰武大数据产业集聚区、宝厦集成建筑基地、徐工二期、浙江玉环汽摩配等项目建设步伐。加快推进环保产业园30万吨/年废铅酸蓄电池综合回收利用等4个项目开工建设。推动北京牛栏山二锅头、上海三爱富化工、彰武年产20万吨乙醇、三一重工产业园等项目落地。围绕特色资源、产业基础等比较优势，盘活低效用地、工业厂房、存量资产，延伸产业链条，包装储备一批科技含量高、税收贡献大、质量效益好的项目。侯卫东官场笔记

紧紧抓住新一轮东北振兴、国家支持资源型地区经济转型、突破辽西北、县域经济发展等重大历史机遇，用好用足各项政策。围绕中央预算内资金、国家重大专项、省产业投资引导基金等政策性资金，积极争取支持，力争更多项目纳入上级盘子。

（二）以改革创新为动力，全面释放发展活力

积极推进“三去一降一补”，坚决淘汰落后过剩产能，巩固去产能成果，建立长效机制，严防死灰复燃。逐步建立多主体供应、多渠道保障、租购并举的住房制度，房地产销售面积增长10%以上。加大对金融机构、重点企业及资本市场排查力度，守住不发生系统性金融风险的底线。继续清理涉企收费，进一步降低企业制度性交易成本和生产要素成本。围绕新兴产业、县域经济等薄弱环节，努力补齐短板。

持续推进“放管服”改革，进一步取消和下放行政职权。建立行政审批事项分类管理制度。深化“双随机、一公开”监管改革，探索推行跨部门联合检查。全面清理、整合、规范各类行政中介服务事项。深化政府机构和事业单位改革。建立公务用车使用管理平台。

深入落实“1+N”系列政策方案，提升交通、城建等企业集团运营水平，加快组建公用事业、医疗、体育、教育等集团步伐。继续推进供热体制、公交体制改革。

实施引进高层次人才三年计划，确保人才引得进、留得住、用得好。培育科技型中小企业20户，实现科技成果转化20项。深化与辽工大市校合作。促进大众创业、万众创新上水平，加快中科院技术转移阜新分中心建设，实施科技创新创业“示范工程”，组建产业技术创新战略联盟。

（三）以招商合作为牵动，提高对外开放水平

继续开展“招商季”活动，坚持全员招商，强化领导带头领任务、走出去，压实各级各部门责任，主动开展协会招商、展会招商、商会招商等活动，形成人人关心、人人参与招商引资的浓厚氛围。完善重点项目专人跟踪服务机制，提升招商成效。

以国内外500强企业、行业龙头企业、上市公司等为目标，积极开展上门招商。积极引进战略投资者，吸引和推动大型央企、省企、民企在阜设立地区总部以及研发中心、营销中心和生产加工基地。围绕重点产业，按照“两头延伸”的原则，大力引进产业关联度高、企业协作性高、产品附加值高的项目。结合产业园区建设，支持龙头企业打造产业集聚园，鼓励企业以商招商。

主动对接“一带一路”，积极参与“五大区域战略”，加快融入辽宁自贸区，建立常态化招商机制。主动承接京津冀、长三角地区产业转移，深化沈阜互动合作，加强与江苏盐城对口合作，搭建平台、建立机制、互动共赢。

（四）以产业园区为载体，培育壮大实体经济

扎实推进“一带五基地”建设。以国家支持资源型地区转型发展为契机，积极融入全国重要经济支撑带建设；以液压和铸造产业为依托，积极融入先进装备制造业基地建设；以保利智能化和航天神坤项目为抓手，积极融入重大技术装备战略基地建设；以煤化工和氟化工产业为支撑，积极融入国家新型原材料基地建设；以实施新一轮示范带建设三年滚动计划为载体，积极融入现代农业生产基地建设；以深化高校院所产学研用为牵引，积极融入重要技术创新与研发基地建设。

优先发展农产品加工业，发挥粮食基地、品牌集聚等优势，发展玉米、花生等深加工项目，促进农产品加工业向食品工业转变。加快能源产业向清洁化、再生化发展，巩固风电、扩大光伏。抢抓军民融合发展重大战略机遇，深化与军工企业对接、合作。加快传统产业改造升级，培育战略新兴产业，大力发展特色产业。

创新园区管理体制，推行“管委会+公司”模式，实现扁平化管理。深化与中科院、国家建材规划院、浙江大学等机构合作，提升研发检测平台水平。创新园区融资模式，进一步完善园区基础设施。

制定政策，分类指导，因企施策，促进“小升规”“个转企”“大做强”，全年规上企业达到240户。支持现有企业做强做大，现有企业新上项目、扩大规模同等享受招商优惠政策。鼓励引导本市关联企业间、市矿企业间的产品协作配套。支持阜矿集团多元化发展。加强对纳税前100名重点企业的运行监测。弘扬工匠精神、劳模精神和企业家精神，营造企业家健康成长环境。

（五）以市场需求为导向，稳步提升服务业质量

加快现有商贸、住宿、餐饮等传统服务业的改造升级，积极发展新业态。扩大6个省级服务业集聚区经济效益，营业收入增长10%。加快兴隆CBD三期、万达广场、红星美凯龙、义乌小商品城等项目建设。积极发展城区经济，提高服务业在城区经济中的比重。办好房交会、玛瑙博览会、美食节等展会，培育消费热点。

推动文化、旅游融合发展，抓好世界玛瑙之都、禅修温泉、北方文化名山、国家矿山公园、沙地国家森林公园、黄家沟生态旅游度假区六大旅游区建设。发展生产性服务业，加快中国邮政集团物流中心、新邱煤炭物流园等项目建设。引进2家金融机构，推动金控公司全面运营，增强金融服务实体经济能力。加快阜新银行、金凯化工上市，新培育一批潜力型企业筹备上市。

积极培育服务业增长点。围绕阜新进入高铁时代，规划一批休闲旅游、医养结合等服务业项目。大力发展外包服务业，扩大阜新产品网上销售规模，推进线上线下融合发展。主动融入辽宁与阿里巴巴、京东商城等战略合作项目，发展光华科技等阜新特色平台。

围绕“一带一路”战略，培育更多出口企业，提升出口质量和附加值，外贸出口总额实现15.8亿元、增长5%。

（六）以富民强县为抓手，提升现代农业水平

加快实施突破辽西北现代农业三年攻坚方案，巩固提升60万亩，新建30万亩。调减玉米种植面积10万亩，尽快实现农业由总量扩张到质量提升的转变。组织实施旱地农业发展规划，加快建立避灾抗灾长效机制。突出农业绿色化、优质化、特色化、品牌化，组织行业协会和企业，培育更多阜新特色产品。实施农业产业化龙头企业提升工程，促进伊利牧场等一批项目落地。坚持种养结合、农牧结合，丰富畜牧产业链条，新建改扩建标准化养殖小区10个。严格落实粮食安全省长责任制有关工作要求，大力实施“优质粮食工程”。

深入实施县域经济一二三产业链接计划，推动三次产业融合发展。坚持工业发展园区化，加快培育壮大农产品加工、粗铜加工、机械配套、高端建材、铸造、玛瑙、硅砂、蒙医药等县域重点产业。加快县域服务业集聚区建设，支持县城及中心乡镇商业发展，培育一批新增长点。

实施县乡财政管理体制改革，全面启动县乡财政“分灶吃饭”。巩固农村土地确权工作成果，推进承包地“三权分置”改革和互换并地，促进土地流转。大力推进“公司+合作社+农户”模式，引导工商资本下乡，培育家庭农场、农民合作社等新型经营主体。深化国有林场、农垦等改革，提高农业保险水平。

实施乡村振兴计划，鼓励发展“一乡一业”“一村一品”。加快培育国家级和省级特色乡镇。实施农村集体经济发展壮大计划，促使生产方式由土地耕作向土地经营转变，培育一批特色产业突出的乡村。支持引导大学毕业生、外出务工农民等各类群体返乡创业，培育树立一批发展带头人。实施“厕所革命”，全力推进宜居乡村建设，加强垃圾排放管控、畜禽粪便治理、秸秆综合利用，全面改善农村生态环境、人居环境和发展环境。

（七）以生态建设为重点，打造宜业宜居城市

拆除燃煤锅炉30台，淘汰黄标车及老旧车500辆，有效防控秸秆焚烧。全年空气质量达标率达到80%以上。积极推进海州露天矿环境治理。强化污染源监管，依法清理饮用水源保护区内违章建设项目，加快细河全流域治理提升、城市防汛“1+3”等重点工程建设，确保各类污水处理设施稳定运行，实现细河水质稳定达标。按时保质保量完成中央环保督察整改任务。

启动全国第二次污染源普查工作，建立健全重点污染源档案、污染源信息数据库和环境统计平台。全面划定并严守生态保护红线。依托“三北”防护林、防沙治沙等重点工程，完成人工造林9.6万亩。继续加强水土保持生态建设。

采用网格化城市管理模式，实行精细化管理。以启动创建国家级卫生城市和文明城市为契机，贯彻市容和环境卫生管理条例，重点抓好老旧小区改造、垃圾收集清运、人行步道维修、城市绿化和水系管理，实施小街背巷路灯安装和维修工程；大力弘扬社会主义核心价值观，培育城市文化，提升群众生活品质。完成高铁阜新站前基础设施建设，积极推进京沈高铁阜盘联线列入规划。加快推进阜奈高速、“锦阜高”铁路扩能改造第二段工程建设。

（八）以保障和改善民生为目的，努力增进百姓福祉

各位代表，打造人民满意政府，必须坚持以人民为中心，切实解决人民群众最关心、最直接、最现实的问题。今年市政府重点办好20件民生实事。

1.16266名建档立卡贫困人口稳定脱贫、48个贫困村销号，阜蒙县贫困县“摘帽”。

2.维修改造老旧小区29个，改造老旧小区居民室内供水设施1.5万户、供水管网10公里，改善居民生活环境。

3.市妇女儿童活动中心投入使用。

4.维修改造老城区39条街路和部分人行步道、健康步道，打通红光路等街路，新增街巷路灯500基，方便市民出行。

5.新建和改造城区垃圾清运站20座，新增和更新环卫设施设备600台套，打造干净整洁城市环境。

6.增加城区新能源公交车数量，率先在全省建成智慧公交。

7.改造农村厕所6870座，新建城区公共厕所30座。

8.改造棚户区2346套，改造农村危房1289户，为3250套廉租房发放补贴，解决部分低收入家庭住房难问题。

9.实施就业技能、职工职业技能培训，全年新增城镇就业1.6万人。

10.提高高龄产妇生育保障能力，全面落实“二孩”政策。

11.支持少数民族特色村镇、文化产业、优势产业、改善民生等项目20个，促进民族地区发展。

12.改造薄弱学校17所，维修校舍10万平方米，新建改扩建幼儿园6所，改善办园条件18所，提升教育教学保障水平。

13.以“玉龙路、高林台河、九营子河”为重点，建设环城休闲绿化带，城区植树8万株，提高城市绿化水平。

14.推进医院“分级诊疗”，实行医保支付按病种分值结算，实现省内异地安置人员就医一卡通，缓解看病难、看病贵问题。

15.完成中央及中央下放企业“三供一业”维修改造工作。

16.提高城乡低保和特困人员救助标准，建设15个街道（乡镇）社区服务中心。

17.依托互联网，建立集社区、交通、行政审批等于一体的警务综合服务体系，方便群众办事。

18.开展运动健身大讲堂、篮球大篷车、国民体质监测等活动，提高市民健身意识。

19.深化领导包产业、包企业、包项目机制，实施精准帮扶，为企业提供优质服务。

20.全面推行“9110”改革，放大“3550”成效，促进行政审批便民提效。

通过严控新增、债券置换、强化预警等措施，有效防范和化解政府债务风险；通过抓好保险征缴、社保扩面、基金管理等工作，化解企业养老金风险缺口；通过加强全链条监测、加快不良贷款处置、支持企业债转股等方式，确保金融风险总体可控。协调推进民族宗教、妇女儿童、职工、老龄、残疾人等事业健康发展。争创新一轮全国双拥模范城。深入开展“七五”普法工作。做好第四次全国经济普查。以让全市人民吃得放心为目标，实施食品安全提升工程。构建和谐劳动关系，加大劳动监察执法力度。推进平安阜新建设，加强社会治安防控，提高应急管理能力；全面落实安全生产各类责任，严防重特大事故发生；积极化解各类矛盾纠纷，保持社会和谐稳定。

（九）以制度约束为根本，打造一流营商环境

坚持问题导向、制度先行，开展“优化营商环境推进年”活动，真正让企业进得来、留得住、干得好、做得强。

认真学习借鉴沈阳等地先进经验，结合阜新实际，进一步优化审批流程、压缩审批时限、精简审批手续，全面实施“多规合一”“多评合一”“多证合一”改革。

巩固“三大整顿”成果，深刻汲取“10•18”事故教训。深入开展政务环境、执法环境、招商环境和市场准入、违规增加企业负担、小微企业融资难等方面专项整治。健全多层次监督体系，变“被动服务”为“问需服务”。

支持民营经济发展，努力破除歧视性限制和各种隐性障碍，构建“亲”“清”政商关系。加强对营商环境投诉的受理、办理力度，及时发现问题、解决问题，完善企业服务直通车等机制，确保向企业承诺的事马上就办，办就办好。

（十）以人民满意为标准，加强政府自身建设

以党的建设为统领，将党建工作与中心工作相互融合，通过党建强班子、促实干，提升政府工作效能，提高公务员队伍素质，建设人民满意的政府。

打造服务型政府。认真开展“不忘初心、牢记使命”主题教育活动，深入推进“两学一做”学习教育常态化制度化，打造一支忠诚、干净、担当的公务员队伍。全面履行“一岗双责”，实施加强党的机关和基层组织建设三年计划，提升政府机关党建水平。加强意识形态工作，弘扬社会新风尚，传递社会正能量。加快推行“互联网+政务服务”，完善网上审批、公共资源交易、市民综合诉求三大平台，及时回应群众关切，提升服务质量。

打造高效型政府。坚持精细化管理，努力做到程序规范化、流程制度化、工作项目化，建立工作台账，实行销号办理，确保各项工作时间清、任务明、标准细。坚持目标导向，推进行政审批事项少、流程优、服务好、成本低、效率高。健全督查问责机制，坚决整肃庸政懒政怠政行为。健全激励机制和容错纠错机制。加强政府“智库”建设，不断提高政府决策科学化水平。

打造实干型政府。坚决贯彻落实国家和省决策部署，做到市委有决策、政府有举措、落实见成效。坚持一级带着一级干、一级做给一级看，带动政府系统广大干部真抓实干。保持艰苦奋斗、戒骄戒躁的作风，培养专业能力、专业精神，积极走出去，招商引资、学习先进；主动走上去，争取支持、掌握信息；经常走下去，调查研究、了解实情。围绕全年目标，列出时间表、任务书、路线图，挂图作战、一抓到底。

打造法治型政府。坚持向市委报告工作制度，自觉接受人大依法监督和工作监督、政协民主监督，认真办理人大代表建议和政协委员提案。深入推进依法治市，全面推行政务公开，完善政府投资类项目程序，提高政府的执行力和公信力。把党内监督同国家机关监督、司法监督、群众监督、舆论监督贯通起来，实现监察监督全覆盖，确保权力在法治轨道上正确运行。

打造廉洁型政府。实施加强党风廉政建设三年计划，严格遵守党章党规党纪，严格落实中央八项规定精神及省市委实施细则，抓好省委巡视反馈整改。全面加强重点领域审计监督。驰而不息推进作风建设，坚决克服形式主义、官僚主义10种作风顽疾，真正做到干部清正、政府清廉、政治清明。

各位代表，蓝图已经绘就，奋斗创造辉煌。让我们紧密团结在以习近平同志为核心的党中央周围，高举习近平新时代中国特色社会主义思想伟大旗帜，在市委的坚强领导下，承前启后，继往开来，以时不我待、只争朝夕的精神状态，奋力走好新时代阜新转型升级之路，谱写新时代阜新改革开放和现代化建设的新篇章！
